# Virtual Reality as a Moderator of Psychedelic-Assisted Psychotherapy

**DOI:** 10.3389/fpsyg.2022.813746

**Published:** 2022-03-04

**Authors:** Agnieszka D. Sekula, Luke Downey, Prashanth Puspanathan

**Affiliations:** ^1^Centre for Human Psychopharmacology, Swinburne University of Technology, Hawthorn, VIC, Australia; ^2^Enosis Therapeutics Pty. Ltd., Melbourne, VIC, Australia; ^3^Institute for Breathing and Sleep, Austin Hospital, Melbourne, VIC, Australia

**Keywords:** altered state of consciousness, MDMA, psilocybin, set and setting, integration, psychedelics, virtual reality, psychotherapy

## Abstract

Psychotherapy with the use of psychedelic substances, including psilocybin, lysergic acid diethylamide (LSD), ketamine, and 3,4-methylenedioxymethamphetamine (MDMA), has demonstrated promise in treatment of post-traumatic stress disorder (PTSD), anxiety, addiction, and treatment-resistant depression. Psychedelic-assisted psychotherapy (PP) represents a unique psychopharmacological model that leverages the profound effects of the psychedelic experience. That experience is characterized by strong dependency on two key factors: participant mindset and the therapeutic environment. As such, therapeutic models that utilize psychedelics reflect the need for careful design that promotes an open, flexible, trusting mindset and a supportive setting. To meet this need, the PP model is increasingly supplemented by auxiliary methods, including meditation, relaxation, visualization or spiritual practices. We suggest virtual reality (VR) as a full-spectrum tool able to capitalize on and catalyze the innately therapeutic aspects of the psychedelic experience, such as detachment from familiar reality, alteration of self-experience, augmentation of sensory perception and induction of mystical-type experiences. This is facilitated by VR’s evidenced capacity to: aid relaxation and reduce anxiety; buffer from external stimuli; promote a mindful presence; train the mind to achieve altered states of consciousness (ASC); evoke mystical states; enhance therapeutic alliance and encourage self-efficacy. While these unique VR features appear promising, VR’s potential role in PP remains speculative due to lack of empirical evidence on the combined use of VR and PP. Given the increased commercial interest in this synergy there is an urgent need to evaluate this approach. We suggest specific VR models and their role within PP protocols to inspire future direction in scientific research, and provide a list of potential disadvantages, side effects and limitations that need to be carefully considered. These include sensory overstimulation, cyber-sickness, triggering memories of past traumatic events as well as distracting from the inner experience or strongly influencing its contents. A balanced, evidence-based approach may provide continuity across all phases of treatment, support transition into and out of an ASC, deepen acute ASC experiences including mystical states and enrich the psychotherapeutic process of integration. We conclude that the potential application of VR in modulating psychedelic-assisted psychotherapy demands further exploration and an evidence-based approach to both design and implementation.

## Introduction

### Treatment Using Psychedelics

Classic psychedelics, such as psilocybin, *N,N*-dimethyltryptamine (DMT) or lysergic acid-*N,N*-diethylamide (LSD), and psychedelic-like substances, such as 3,4-methylenedioxymethamphetamine (MDMA) or ketamine, have a long history of medicinal use (Carod-Artal, 2015; Nichols, 2016). The past 10-years has seen a resurgence in the use of and research into the potential therapeutic benefits of psychedelic compounds (Carhart-Harris and Goodwin, 2017). Psychedelics have primarily been examined as aids to psychotherapy in the treatment of post-traumatic stress disorder (Mithoefer et al., 2019), treatment-resistant depression (Carhart-Harris et al., 2021), and substance dependence (DiVito and Leger, 2020). Controlled administration of psychedelics can be a tool for facilitating psycho-behavioral change (Garcia-Romeu et al., 2014, 2016; Barrett and Griffiths, 2017) and lead to marked, rapid, and enduring benefits in both healthy volunteers and patient populations, particularly when accompanied by a supportive context (e.g., psychological support; Luoma et al., 2020).

The impact of psychedelics upon aspects of mental health and well-being have been attributed to the phenomenological features of the psychedelic experience, in particular peak or mystical states (Griffiths et al., 2006). Those phenomenological features as well as the depth of the mystical experience (ME) are affected by the type of psychedelic drug, dosage, the environment in which it is administered, and participant’s individual predispositions, in particular their openness to the experience (Carhart-Harris et al., 2018). These features, along with the degree to which the psychedelic experience is integrated into the participant’s psyche, are thought to regulate the therapeutic outcomes and emergence of any persisting, positive changes in attitudes and behavior (Haijen et al., 2018).

### Limitations of Psychedelic-Assisted Psychotherapy

Studies on Psychedelic-Assisted Psychotherapy (PP) are yet to systematically evaluate the role of context. Whilst it remains unclear what the most favorable contextual elements are, both personal consumption and psychedelic retreats favor nature-immersion models (Kettner et al., 2021; Ruffell et al., 2021), that were promoted by early researchers in the field (Hofmann and Ott, 1980), in contrast to clinical settings in use today. Nature-immersion models aim to maintain a continuous, relaxed, multi-sensory engagement throughout the psychedelic journey (Shanon, 2002; Winkelman, 2021). The clinical approach, largely due to logistical limitations, departed from this model, resulting in segmentation into distinct, separate phases of treatment (preparation, dosing, and integration), which could impede a fluid transition throughout PP. Moreover, although study methods in clinical or research settings are carefully designed in accordance with principles of the *set* and *setting* protocol (Johnson et al., 2008; Hartogsohn, 2017), there is often a lack of congruence between the dosing session, and the preparation and integration sessions (Griffiths et al., 2016). Within individual trials, preparation and integration phases may be based on diverse psycho-therapeutic frameworks, including the change of the role of the therapist from that of supporting facilitator to that of psychotherapy provider (Johnson et al., 2014) or the involvement of different therapists (Fuentes et al., 2020). This discontinuity is further perpetuated by different phases taking place in different environments (e.g., Griffiths et al., 2016) and the variable use of diverse aids, particularly music, which plays a critical role throughout the dosing session but is not always formally utilized during integration sessions (Kaelen et al., 2018). Moreover, profound psychedelic experiences present challenges for the process of integration, due to their ineffable nature (Neitzke-Spruill, 2019), impaired perception of time (Wittmann et al., 2007; Wackermann et al., 2008; Yanakieva et al., 2019), the vast number of thoughts experienced (Carhart-Harris et al., 2016), overwhelming emotions (Gouzoulis-Mayfrank et al., 1998) and impaired recall even shortly after (Barrett et al., 2018; Doss et al., 2018b). This recall may be further impacted by an internal resistance to reliving challenging or highly emotionally loaded experiences in the absence of the psychoactive substance. It is also recognized that traditional psychotherapeutic approaches may not be best suited to therapeutic modalities that depend on intentional induction of a psychedelic-like state. Previously auxiliary practices such as somatic therapy, breathwork or mindfulness, are increasingly moved to the forefront of the therapeutic process (Payne et al., 2021).

Considering that each phase of treatment impacts the course and the outcome of PP, the lack of congruence throughout treatment (e.g., Carhart-Harris et al., 2018), could be postulated as a causative factor in reported psychological side effects such as paranoia or violent urges (e.g., Studerus et al., 2011; Johnstad, 2021) and waning of positive outcomes with time (e.g., Bogenschutz et al., 2015). It is argued that adopting a more unified structure, ensuring perceived continuity across all phases of treatment, could minimize psychological side effects and sustain positive outcomes of PP for longer. A more congruent design is also expected to encourage an overlooked phase of the psychedelic journey, which may follow the integration phase: *self-practice*. A clear, uninterrupted structure of PP paves the path for participants to continue integration privately, at their own pace, for as long as it is of benefit to them.

### Virtual Reality Therapy

The psychedelic state is characterized by a non-ordinary experience of consciousness, which involves subjective changes of attention, awareness and/or affect. Therapeutic applications of non-ordinary states that are similar to those induced pharmacologically with psychedelic drugs, can also be evoked physically (e.g., through shamanic dance; Lee et al., 2016), psychologically (e.g., through meditation; Hanley et al., 2018), or through sensory stimulation (e.g., light flicker; Bartossek et al., 2021). Virtual Reality (VR) has also emerged in recent times as having the capacity to disrupt the rigidity of typical conscious experience (Glowacki et al., 2020). Typically, this involves providing specifically designed visual content, viewed by a participant through a head-mounted display, which may also include auditory components and less frequently other sensory input such as haptic feedback (Repetto et al., 2009; Le May et al., 2021). In this way, VR can be used to alter the sensory experience of users, including evoking particular mental states and emotional responses, for example the sense of awe (Stepanova et al., 2019). It is also possible to simulate visual hallucinatory experiences (Suzuki et al., 2017). VR’s capacity to transiently alter a participant’s perspective and disrupt the rigid patterns of mental experience has also found application in the treatment of a variety of mood disorders, often alongside traditional therapies (Fernández-Caballero et al., 2017).

Virtual reality experiences are characterized by several distinct features, which have been evidenced to confer substantial benefits to patients undergoing various forms of psychotherapy (e.g., Boeldt et al., 2019), including augmentation of the sense of presence in exposure treatment (e.g., Rothbaum et al., 2001) and acute stress relief in treatment of anxiety (Tarrant et al., 2018; Donnelly et al., 2021). VR’s capacity to distract from external cues has been utilized in pain reduction, both as an adjunct to pharmacotherapy, most commonly with opioids (Das et al., 2005), and as its replacement (Schmitt et al., 2011). VR exposure therapy has also been explored in combination with other pharmaceuticals, including cortisol in treatment of acrophobia (Dominique et al., 2011), cognitive enhancers (yohimbine hydrochloride) in treatment of aerophobia (Meyerbroeker et al., 2012) and antidepressants (paroxetine or venlafaxine) in treatment of agoraphobia (Castro et al., 2014).

Whilst these two different therapeutic approaches have developed separately, but in parallel, consideration has recently been given to a composite approach in order to enhance efficacy (Moroz and Carhart-Harris, 2018). For example, it has been suggested that VR might be used to optimize the environment in psychedelic sessions (Aday et al., 2020). Despite the growing interest in combining VR and psychedelics, which is emerging predominantly within the commercial industry, there are no experimental studies to date which investigate this combined approach. Nonetheless, a large body of research on VR and the growing body of research on therapeutic application of psychedelics allow for a theoretical, speculative investigation of the features of VR that are relevant to an altered state of consciousness associated with psychedelic use. Here, unique features of VR and their functional relationship with psychedelics are discussed, followed by a theoretical exploration of the range of synergistic outcomes that could be expected from simultaneous application of VR therapy and PP. To encourage an empirical exploration, we provide specific examples of VR models that could be integrated into various PP protocols, considering potential advantages against substantial limitations and possible disadvantages of this synergistic approach.

## Features of Virtual Reality Relevant to Psychedelics

### Relaxation

Treatments of affective disorders (e.g., PTSD, obsessive-compulsive disorder, addiction) frequently involve relaxation methods for their significant benefits in reducing anxiety and depression symptoms (León-Pizarro et al., 2007; Manzoni et al., 2008), improving physiological markers of the stress response (Esch et al., 2003) and decreasing physical discomfort (León-Pizarro et al., 2007). Relaxation techniques have also been observed to increase patients’ willingness to continue treatment (Pasyar et al., 2015; Ream et al., 2021) and to ensure extension of treatment benefits long term (Libo and Arnold, 1983).

A state of relaxation before psychedelic drug administration is one of the main predictors of positive self-dissolution (Dittrich, 1998), underscoring the importance of methods and environments that promote a relaxed mindset before and during psychedelic treatment. Despite the common use of mindfulness, breathwork or visualization techniques during PP (Watts and Luoma, 2020), stress and anxiety before or during dosing remain one of the most commonly reported undesired effects associated with psychedelic use in recreational and therapeutic settings (Griffiths et al., 2006; Studerus et al., 2011; Bienemann et al., 2020). This points to the need for more reliable relaxation methods for PP.

A recent meta-analysis showed that a wide range of VR interventions, in particular when using nature-based audio-visual stimuli, reliably facilitate a state of relaxation (Riches et al., 2021), highlighting VR’s potential for use in conjunction with psychedelic therapy, which can be viewed as challenging by some clients. Although sustained, long term effects of VR relaxation interventions are yet to be ascertained (Riches et al., 2021), VR-based relaxation sessions commonly precede VR exposure treatment (Repetto et al., 2013; Son et al., 2015) due to recognized benefits of VR in acutely reducing distress symptoms associated with anticipation of a stressful or emotionally challenging event (Maples-Keller et al., 2017). VR has also been shown to outperform a diverse range of control conditions as a stress relief intervention in acute settings, including desktop-based relaxation interventions (Liszio et al., 2018), guided meditation and progressive relaxation exercises (Veling et al., 2021). Those results indicate that VR may be suitable to promote the state of relaxation during psychedelic treatment if applied prior to psychedelic exposure, and to relieve stress acutely if it arises during the session.

### Buffering

The efficacy of VR as a mental health treatment modality can be partially attributed to its capacity to act as an “immersive distractor” (Le May et al., 2021). This ability to detach the participant from other sensory experiences could explain, for example, why VR leads to significant alleviation of pain in acute (Hoffman et al., 2008; Maani et al., 2011) and chronic conditions (Schmitt et al., 2011). Therefore, whilst VR is recognized predominantly for its ability to provide sensory stimulation, its therapeutic utility appears to be equally dependent on its ability to subtract it.

Psychedelic-assisted psychotherapy relies strongly on maintaining an appropriate balance between sensory enrichment and sensory deprivation (Carhart-Harris et al., 2018). For example, application of blindfolds in combination with music, and occasional use of visual cues like nature books, are used to modulate the sensory input (e.g., Johnson et al., 2014), although blindfolds only allow limited, binary control over environmental stimuli (no visual stimuli vs. complete visual stimuli). Moreover, despite recognition of the importance of environmental design in curating the psychedelic experience, studies that utilize psychedelic substances rarely exploit the potential of using the environment to create a distinction between the day-to-day and the ethereal (Fuentes et al., 2020). Clinical and retreat environments are filled with familiar, even domestic cues, which may keep the attention rooted in the mundane, hijacking the experience instead of inspiring deeper emotional processes (Hartogsohn, 2017). What is more, those cues are likely to be perceived differently by the therapist and the patient who is under the effects of a psychedelic, forming a wedge in perceptual congruence and in turn perhaps, a barrier to therapeutic alliance. Finally, current approaches to setting design have not seen major innovation since the earliest psychedelic trials (Grinspoon and Bakalar, 1979).

In contrast to the current binary approach, VR allows fine control over the context and richness of stimuli that is provided. The virtual world can reduce distraction from the real world by replacing familiar cues with unfamiliar, fantastical objects and symbols (Quesnel and Riecke, 2018), that are conducive of deep immersion into the psychedelic experience (Glowacki et al., 2020). VR can therefore be used to transport the user out of the treatment environment and into an alternate reality, acting as a buffer zone, which is equally novel for both therapist and participant, facilitating awareness of the immediate experience in a more mindful way.

### Mindful Presence

The intensity of the psychedelic experience, especially the experience of ego dissolution, can sometimes prove difficult and patients may resist the effects or attempt to temper them, for example by using engagement with the therapist as a distraction (Bourzat and Hunter, 2019). On the other hand, staying engaged despite the discomfort and surrendering to experiences that are challenging seems to have a beneficial impact on therapeutic outcomes (Johnstad, 2021). The capacity to stay engaged with a challenging sensation may be strengthened by learning to deepen the sense of presence without judgment, a skill that is commonly practiced by mindfulness meditators (Baer, 2015).

Mindfulness meditation is a technique that aims to expand the sense of awareness of diverse physiological and psychological sensations, without assessment or attachment to any of them (Kabat-Zinn, 2009). When used in combination with psilocybin, mindfulness practice has led to a deepening of the acute psychedelic experience, in particular a stronger sense of unity, bliss and spirituality (Smigielski et al., 2019a) and facilitated lasting therapeutic outcomes, including positive changes in psycho-social functioning (Griffiths et al., 2018; Smigielski et al., 2019b). Nonetheless, mindfulness is a challenging practice that requires time and commitment, and clients may still be unsuccessful in reaching the mindful state even when both are abundant (Hunt et al., 2020).

Mindfulness interventions are gaining increasing popularity among psychedelic practitioners (Payne et al., 2021) and VR has proven successful in helping people engage with mindfulness as well as reach the meditative state (Perhakaran et al., 2016; Seabrook et al., 2020). VR-based meditation interventions lead to a deep state of mindfulness, that has been reflected in changes of EEG patterns indicative of a relaxed, meditative state. Those changes, for example a shift of high to low beta frequencies and reduced beta activity in anterior cingulate cortex (Tarrant et al., 2018), were not observed in control conditions such as guided audio meditation (Yildirim and O’Grady, 2020). Significant increase in state mindfulness and positive affect have also been observed following VR mindfulness sessions in both clinical (Navarro-Haro et al., 2019) and non-clinical settings, making it suitable for all types of therapeutic environments that employ psychedelic substances. Technology could help mitigate the main barriers to drawing benefits from a mindful attitude in psychedelic treatment, predominantly the discouragement caused by uncertainty of achieving sufficient depth of mindfulness, and the need for often unrealistically lengthy, challenging training.

### Augmenting Peak States

In approaching the psychedelic experience, a relaxed, mindful mindset has been observed to reduce the likelihood of adverse psychological events (Johnson et al., 2008) and is conducive to producing a mystical experience (Smigielski et al., 2019a). The depth of the ME, commonly measured by the mystical experiences questionnaire (MEQ30), is one of the key predictors of positive outcomes following psychedelic psychotherapy (e.g., Garcia-Romeu et al., 2014), including a decrease of clinical symptoms and lasting improvements in well-being (Haijen et al., 2018). Although the nature of the ME remains enigmatic, researchers have managed to identify certain phenomenological characteristics, or dimensions, of those experiences that resonate with the majority of participants (Barrett and Griffiths, 2017). These include: the sense of awe, unity and sacredness; timelessness and spacelessness; ineffability; as well as a sense of authenticity and validity of the reality that is being witnessed, despite its unrecognizable, sometimes even bizarre makeup (Hood, 2001; MacLean et al., 2012).

Highly immersive VR experiences also demonstrate some of those same, unique characteristics. The loss of sense of space, time and even connection to one’s body is a commonly reported phenomenon (Seabrook et al., 2020). Patients tend to lose points of reference with reality, which seems to engage them in the process of contemplation and introspection (Seabrook et al., 2020). The resultant absorption into VR can counteract reality-testing mechanisms, tricking our senses into making a surreal scenario appear real (Suzuki et al., 2017). Witnessing and believing this alternative reality can lead to a sense of surrender (Seabrook et al., 2020), resulting in a level of engagement with VR scenarios, which can be stronger than that with the real world (Kotler and Wheal, 2017). Additionally, the experience of an alternative reality can also be ineffable (Glowacki et al., 2020).

Virtual reality-induced awe also appears to share functional characteristics with psychedelic mechanisms (Hendricks, 2018). Recent findings on phenomenological aspects of awe distinguished between the immediately available but less impactful “quick boiled awe,” and the deeper, if more difficult to achieve, “slow simmer awe” (Chirico and Gaggioli, 2018). VR-based experiential approach allows for eliciting the latter, more intense form (Gallagher et al., 2015; Chirico et al., 2016, 2017), which has also been proposed as a putative mechanism of psychedelic effects (Hendricks, 2018). VR, in evoking the challenging, destabilizing and deep sensations associated with awe, could act as a medium for a self-transcendental experience (Chirico and Gaggioli, 2018).

Although psychedelic compounds have been used historically and in the present to successfully evoke MEs (Griffiths et al., 2006) and emerging evidence shows that VR can also be successful in eliciting such mystical-type experiences (Glowacki et al., 2020), little empirical work has been performed on joint application of both methods acting synergistically to deepen the mystical state. Psychedelics have previously been combined with a range of other state altering methods, in order to deepen the ME, for example *via* the use of LSD with sensory deprivation (Lilly, 1990), psilocybin with meditation (Smigielski et al., 2019a,b), or hallucinogenic Datura plant with trance inducing dance practices (Du Toit, 1977). Results of those studies suggest that combining different ego-dissolving or awe-inspiring practices may lead to an augmented, cumulative effect in producing a more “complete mystical experience” or “complete ego dissolution,” which are known to play an important role in the healing process (Haijen et al., 2018). Given the wide range of shared properties that are relevant to evoking a ME, the pairing of VR and psychedelics presents itself as a promising candidate for further exploration of this capacity for augmentation.

### Altered States of Consciousness Priming

MEs are preceded and facilitated by unique changes in consciousness that are often referred to in literature as altered states of consciousness (ASC; Vaitl et al., 2005). An ASC is a phenomenological experience characterized by changes in perception of time, space and even oneself, accompanied by a non-ordinary range of attention, awareness and emotion (Ludwig, 1966). Here we refer to ASCs that are subjectively experienced as a form of expansion or transcendence of normal, wakeful consciousness, and are purposefully induced by a consumption of a psychedelic compound (Metzner, 1994), and also, although less reliably, through meditation (Shapiro, 2009; Stapleton et al., 2020), trance (Glicksohn and Ohana, 2011), or practices that induce flow states (Csikszentmihalyi and Nakamura, 2018), among others.

Interestingly, evidence suggests that the ability to achieve an altered state of consciousness can be improved through practice (Kotler and Wheal, 2017). Brain imaging outcomes revealed that experienced meditators exhibit better control over their attentional resources (Farb et al., 2007; Hasenkamp and Barsalou, 2012). This has been attributed to lasting changes in functional connectivity between brain regions responsible for attention and self-awareness (e.g., between the right insula and the prefrontal cortex) that seem to be the result of mindfulness practice (Farb et al., 2007; Hasenkamp and Barsalou, 2012). More experienced meditators are also more likely to experience ego dissolution, which is a hallmark of high dose psychedelic experiences (Hölzel and Ott, 2006; MacLean et al., 2012). What is more, practicing the ability to attain an ASC seems to be translational across different techniques. This is exploited, for example, in the training of American Navy SEALs, who practice common ASC methods (meditation, sensory deprivation, or trance) to optimize their ability to enter a state of flow during missions (Kotler and Wheal, 2017). In a similar way, prior mindfulness meditation practice significantly increases the depth of the ME induced by psilocybin, particularly the dimensions of introvertive mysticism and oceanic boundlessness (Smigielski et al., 2019a), and increases positive emotions associated with psilocybin-occasioned ego-dissolution. Finally, the incidence and the intensity of ME have been shown to be influenced by a clear intention for a mystical or a spiritual experience; this intention can be strengthened with practice (MacLean et al., 2011; Haijen et al., 2018).

The previously presented evidence suggests that practicing the ability to achieve an ASC using one method may increase the capacity for achieving it with other methods. Since early on in its inception, VR has been recognized as a tool capable of eliciting ASCs (Glicksohn and Avnon, 1997), and has been used extensively in the entertainment industry to produce an entire range of ASCs (Weinel, 2018). Moreover, VR has already been highly successful in facilitating other therapies that entail the production of ASC, for example meditation (Perhakaran et al., 2016; Seabrook et al., 2020). Research has also shown that VR can be used to induce visual alterations that closely resemble those brought on by classical psychedelics (Suzuki et al., 2017). It has been suggested that using VR to prepare clients for perceptual alterations induced by psychedelics may have beneficial effects on their expectation of hallucination effects and on pre-trip anxiety (Aday et al., 2020). By providing a comfortable, reliable and potent rehearsal space, VR can equip clients with a sense of awareness of initiating and engaging with an ASC, encouraging a more complete surrender to and exploration of the psychedelic state.

### Therapeutic Alliance

A comfortable and productive relationship between the client and the therapist, known as positive therapeutic alliance, can account for as much as 30% of the positive outcomes of psychotherapy (Hubble et al., 1999). Its value may be even higher for treatments that use psychedelics, due to the palpable impact that setting (including the therapist) has on the hallucinogenic experience (Johnson et al., 2008). Current manuals for psychedelic practitioners agree that establishing successful rapport is the therapist’s primary responsibility (Guss et al., 2020). Moreover, it is essential for that rapport to be established collaboratively (Greer and Tolbert, 1998). Conversely, inadequate therapeutic alliance has been suggested as one of the important factors in participants’ poor response to psychedelic-based treatment (Nielson and Guss, 2018) and may increase the likelihood of adverse psychological reactions during the dosing session (Johnson et al., 2008).

Despite the common argument that use of technology in therapy can reduce the human connection (Przeworski and Newman, 2012), VR therapies are reported to have one of the highest therapeutic alliance rates (e.g., Garcia-Palacios et al., 2007; Meyerbröker and Emmelkamp, 2008) and satisfaction rates (Beck et al., 2007; Baños et al., 2009) across various therapeutic approaches. Relaxing VR environments reinforce patient involvement in treatment and increase positive rapport (Boeldt et al., 2019). VR is especially effective in facilitating a sense of safety and trust among the most traumatized or apprehensive patients, who tend to choose VR over talking to a counselor in person in order to work through their traumatic experiences (Garcia-Palacios et al., 2007; Wilson et al., 2008). Lastly, VR scenarios can be designed, by way of interactivity, to give patients an element of control and ownership over their surroundings, and as a result a sense of agency within the experience (Ewalt, 2018). The resultant collaborative exploration of this alternative world may be able to foster a better sense of equality by disrupting the therapist-patient hierarchy (Wrzesien et al., 2011).

### Self-Efficacy

One of the key roles of preparation sessions in PP is to promote a non-avoidant attitude, particularly to challenging components of the psychedelic experience (Mithoefer et al., 2008; Davis et al., 2020). A mindset that welcomes challenging thoughts and emotions, rather than avoiding them, determines the depth of the psychedelic experience and subsequently its therapeutic impact (Studerus et al., 2011; Carbonaro et al., 2016; Haijen et al., 2018; Roseman et al., 2018).

VR has been shown to help patients overcome the fear of sharing their deepest, most disturbing thoughts, even when they are unwilling to discuss them directly with their therapist (Maples-Keller et al., 2017; Prudenzi et al., 2019). Following a single VR thought-defusion session, clients reported significant improvement in negativity, conviction and discomfort related to a patient-specific disturbing thought (Prudenzi et al., 2019). VR therapies are also thought to facilitate an increase in self-efficacy and a decrease in negative self-statements (Meyerbröker and Emmelkamp, 2008), giving patients a sense of agency in coping with negative self-referential thoughts. As such, VR can be used during the preparation phase to strengthen self-efficacy in confronting difficult thoughts or emotions (Premkumar et al., 2021), thus promoting the ability to surrender to challenging experiences as they occur.

## Models of Incorporating Virtual Reality Into Psychedelic-Assisted Psychotherapy

Based on the presented parallels in functional properties of VR and psychedelics, we suggest potential methods of VR application in a PP protocol, which fall into four categories: expansion, transition, cohesion and rescue.

### Expansion

The outcomes of PP capitalize on the combined effectiveness of all its elements, from preparation, through dosing, to integration and beyond; VR is suitable for incorporation into any of these phases.

Firstly, adequate preparation preceding the psychedelic experience is necessary to solidify intention for the upcoming psychedelic journey and prepare clients for unusual psycho-emotional or bodily sensations that may occur (Carhart-Harris et al., 2018). While those sensations are often discussed during preparation sessions, current protocols have limited means of meeting the need for experiential exploration (Guss et al., 2020). To bridge this gap, VR scenarios can be used to practice an ability to achieve an ASC and promote a sense of ease around obtaining and experiencing those states, including but not limited to perceptual alterations (Suzuki et al., 2017). Additionally, VR has been shown to be highly effective in optimizing psycho-emotional processes that also play a role in the phenomenology of the psychedelic state, such as psychological flexibility (Davis et al., 2020; Pinilla et al., 2020; Watts and Luoma, 2020), for example *via* challenging participants to control their impulsivity and sustain attention in gaming environments (Blandón et al., 2016) or by introducing positive changes in the VR’s storyline in response to positive changes in participants’ affect (Cavazza et al., 2014). By repeatedly inducing meditative-like or psychedelic-like states during the preparation phase, for example with hypnotic audiovisual elements or a visually engaging guided meditation program, VR can be utilized to prime occurrences of expanded states of consciousness prior to dosing. After a more relaxed, contemplative or non-ordinary state is reached, VR scenarios can facilitate psychological flexibility training, for example by defusing abstract thought representations of patient-identified barriers, such as loss of control, that could obstruct complete surrender into the psychedelic experience. In turn, this may encourage greater openness in approaching unfamiliar, psychedelic-induced psycho-emotional experiences.

Secondly, the depth and the content of the acute psychedelic experience during the dosing session is of particular importance to the outcomes of therapy (Studerus et al., 2011). When profoundly meaningful, those experiences have been reported to be one of the most or the single most influential experience of one’s life (Griffiths et al., 2006), and have been proposed to have the potential to lead to pivotal changes in one’s feelings, attitudes, behaviors and even personality traits (Brouwer and Carhart-Harris, 2021). Given that psychedelic effects display a remarkable response to external triggers (Kaelen et al., 2018), a supportive, spiritual, or inspiring setting is far more likely to encourage a ME than clinical or laboratory environments (Strassman, 2000; Hartogsohn, 2017). Sensory stimulation with music has also been shown to be capable of evoking meaningful therapeutic experiences during dosing (Kaelen, 2017). The most effective auditory stimuli during the peak phase of the psychedelic experience have been described as homogeneous, with static dynamics, unrecognizable instruments, with regular and consistent phase structure and mostly cyclical compositional form (Barrett et al., 2017). Given the ability of VR to create any environment that can be imagined (Quesnel and Riecke, 2018), its application has unsurprisingly been uniquely successful at creating immersive mystical, spiritual or awe-evoking scenarios (Gallagher et al., 2015). Such models could be harnessed while drug effects last, to construct an optimal setting that increases the chances of inducing a mystical-type experience. On the other hand, any setting provided for the participant during the dosing session may detract from the innately derived healing pathway or even highjack the experience. VR may be best introduced in protocols that already rely on a form of engagement with the external world during dosing, for example protocols that involve nature immersion or group interaction. Within such therapeutic frameworks, VR’s unlimited potential for setting design could be leveraged to provide unique, awe-evoking models, for example, of deep space or unreachable natural landscapes. These could be paired with appropriate auditory stimuli, such as solar system sonification (Tomlinson et al., 2017) or a recording of ocean waves (Erich, 2006), thus unifying the visual and auditory stimuli into a congruent whole. Other designs may include personally meaningful links to tribal or spiritual practices or places of worship, paired with, for example, ritualistic shamanic drumming (Bensimon et al., 2008).

Lastly, the noetic quality of ASC experiences is often at odds with the familiar sensations characteristic of the normal, wakeful consciousness, and is therefore difficult to assimilate into daily life (Kotler and Wheal, 2017), posing a challenge for integration (Guss et al., 2020; Callon et al., 2021). During acute drug effects, communication, especially verbal, can be unappealing, lacking a cohesive narrative, or functionally impossible (Neitzke-Spruill, 2019). Insights that appear obvious and profound during the psychedelic state can also become less clear even shortly after the experience concludes. The ineffable nature of the psychedelic experience may explain why current integration procedures, where recall is based largely on verbal exchange with the therapist (White, 2007), find themselves limited. Instead, the evidence of beneficial outcomes of meditation and spiritual practices on outcomes of PP points to an important role of non-verbal practices in mediating integration (Cohen, 2017; Smigielski et al., 2019a). VR presents as a uniquely suited medium to reliably initiate ASC experiences during the integration process, thus allowing exploitation of their beneficial characteristics, such as broadened awareness or mindful presence (McPeake et al., 1991; Vaitl et al., 2005). If continuity is maintained between VR models used in conjunction with the psychedelic substance and those applied during the integration process, VR may also be able to facilitate greater recall of psychedelic experiences. For example, if a specific natural landscape accompanied by appropriately paired musical sounds (Barrett et al., 2017) is applied during the dosing session, introduction of that same scenario during integration may bring back memories, emotions or insights that were present during the psychedelic experience. Unfortunately, not all reinforcement of memories is beneficial (Doss et al., 2018a,^2020^) and any stimulus that is used to trigger recall of the psychedelic experience puts the participant in danger of having false or negative memories reinforced. Additionally, whilst VR’s capacity to increase recall can be advantageous, it also means that extra care is necessary around its application during integration, to minimize the risk of inducing a vulnerable, challenging or anxious mindset.

### Transition

The effects of most psychedelic drugs do not emerge nor dissipate rapidly, but rather follow parabolic-like changes in intensity (Majić et al., 2015), starting with a slow onset (*pre-peak phase)*, followed by the core of the experience (*peak phase*), and ending with a gradual resolution (*post-peak phase*; (Fox et al., 2018). A latency period follows consumption of a psychedelic substance, after which subjective effects begin to appear, including changes in arousal and perception (Hollister, 1984; Carhart-Harris et al., 2012). Those initial changes may be stressful or anxiety provoking (Hollister, 1984), prompting participants to consciously oppose or subconsciously disengage from deeper immersion into the psychedelic state. This stressful response, together with disruptions in thought that emerge during the pre-peak phase, may also lead to loss of cognitive focus on the intention, which is often essential to reaping full benefit from the remaining part of the experience (MacLean et al., 2011; Haijen et al., 2018). It can be argued that with appropriately designed environments, cues or symbols, VR can gently nudge the attention back to the intention, for example by offering a model that centers around a personalized, patient-designed totem. Such totems can be constructed as a multi-sensory experience, combining visual and auditory elements that are personally significant in an attempt to activate or intensify emotions, thoughts and memories. The audiovisual content may be created to represent a sense of journey, expanding on one of the primary utilities of music in PP, the provision of a sense of guidance (Kaelen, 2017). This process of personalization would play a critical role in accounting for the inter-person variability in individual intentions and in avoiding misdirecting the experience. Nonetheless, such explicit representations may still be inadequate for a construct as ineffable as intention. Therefore, the use of VR during the pre-peak phase may be best reserved for those struggling to utilize their intention as guidance, for example participants who resist the effects of the psychedelic substance or get lost in a cognitive loop. In such cases, VR also offers mild stimulation to maintain alertness and keep focus on the present moment (Seabrook et al., 2020) whilst displacing familiar cues from the treatment setting, that may be distracting or negatively triggering, thus supporting the state of relaxation (Seabrook et al., 2020). Thereby, application of VR during the onset of an ASC can reinforce the intention and encourage a deeper immersion into the psychedelic experience among participants who have previously displayed difficulty in this regard.

When the effects of the psychedelic substance begin to wane, during the so called “resolution period,” psychedelic experiences still echo vividly whilst patients slowly begin to reconnect with the external world (Fox et al., 2018). They may begin to vocalize the contents of their antecedent psychedelic experience in the process referred to as “the first narrative” (Guss et al., 2020), which is often disordered but revelatory for patients. In some models of psychotherapy, the first narrative is explored freely, without explicit guidance from the therapist (Mithoefer et al., 2008; Guss et al., 2020). While holding an open dialogue around the psychedelic experience may present a challenge for therapists (Johnson et al., 2008), a large number of patients naturally gravitate toward VR for its comfortable and less confrontational nature than face to face therapy (Garrett et al., 2017; Riva et al., 2019). What is more, VR provides a wide range of expressive tools to communicate with the therapist, without having to rely on language (Hacmun et al., 2018; Kaimal et al., 2020). Compared to traditional, two-dimensional art, creative expression such as painting, modeling or designing is less inhibited in VR, challenges familiar modes of perception and activates full sensory engagement (Kaimal et al., 2019; King et al., 2019). As such, VR allows a space that can be used to explore and consolidate psychedelic experiences before they need to be shared, for example *via* creating representations of thoughts, insights or emotions with expressive art tools, personalized spatial design of the VR model, or intentional use of symbolism. VR could serve as a physical representation of a memory library, where any information which seems of importance during the resolution period is recorded visually or verbally or both. Such VR models should be personalized and interactive, allow a large degree of flexibility, and ideally be built up over numerous sessions. VR may serve as a blank canvas, with a multitude of available elements that can be used to build personally meaningful contexts. Audio input could be adapted to channel appropriate emotional sentiment, for example grief or hope, drawing on music’s capacity to increase therapeutic engagement by reflecting autobiographical and personally significant content (Kaelen, 2017). Such individually designed models can then be utilized during the integration process, to further explore and discuss the elements that were already established, while adding new insights or actionables, expanding and reconfiguring the memory library throughout the integration phase. However, any tools used to deepen the memory of an experience can affect the process of memory formation and introduce the risk of consolidating a false narrative (Doss et al., 2018b). Other concerns include the following: VR may prove overly stimulating and act as a distractor rather than an aid in recounting or interpreting the ineffable experience; the virtual aesthetic may be at odds with the internal experience and therefore impair the process of self-discovery; predetermined VR input may disengage the participant and deprive the participant of a sense of agency. To mitigate this, VR scenarios used at this point in the dosing sessions should be characterized by a large degree of flexibility and responsiveness, to allow for a participant-driven process of assembling the VR model from within the VR space. VR may best be treated as an advanced tool for self-expression, with the immersive world created by the participant instead of for the participant.

### Cohesion

Therapeutic sessions that revolve around the novel and impactful nature of the psychedelic experience require a clear and receptive mindset, that is in contrast to the occupied and distracted character of day to day cognitive functioning (Davis et al., 2020). Thanks to its unique ability to act as a buffering tool and promote the state of relaxation and mindful presence, VR can aid the process of transition from daily life to the therapeutic setting (Chandrasiri et al., 2020; Seabrook et al., 2020; Riches et al., 2021). Immersive and deeply engaging VR environments isolate one from the distractions of the external world, promote detachment from familiar temporal and spatial reference points, and ground the attention in the here and now (Seabrook et al., 2020). Additionally, contextually rich models that are created in VR can be reapplied in precisely the same manner each time (Maples-Keller et al., 2017), including nuanced factors such as time of day and natural lighting conditions, that cannot be accounted for in real-life environments. Through such repeated use, the sense of presence and relaxation becomes more pronounced and easier to achieve with each session (PeÒate et al., 2008). Scenarios that promote relaxation, mindful presence and buffering often use serene man-made spaces (Järvelä et al., 2021), nature immersion (Liszio et al., 2018), or outer space models (Chirico et al., 2016). These examples are predominantly non-directive, characterized by low to medium intensity stimuli, with wide focal points that require minimal focus. Perceived continuity across phases may be achieved not only by repeated use of the same scenario, but also by the way of a continuous theme (e.g., a forest), repetition of a key object (e.g., a noticeable large oak tree), or the participant playing a familiar role in each experience (e.g., going on a walk in the landscape). These familiar, reliable cues can be returned to at any time (Repetto et al., 2013), also between formal integration sessions (for example during challenging moments when the therapist is not immediately available) or once the integration process concludes, in order to prolong treatment effects *via* self-practice. Ideally, VR scenarios used for self-practice should maintain continuity with VR models used during dosing, whilst building on the goals of therapy. For example the same scenery that was applied during dosing, such as a model of a personally meaningful natural scenery, could be used as a backdrop for an interactive training program that targets a desired behavioral change such as attention training (Li et al., 2020). Interestingly, VR therapies have one of the highest compliance rates (Thielbar et al., 2020) and can significantly improve adherence to other treatments when used as an adjunct to therapy (Navarro-Haro et al., 2019), suggesting that incorporating VR into PP protocols may make participants more willing to continue integration in the form of self-practice. What is more, VR’s capacity to reduce physical discomfort and pain (Hoffman et al., 2011; Garrett et al., 2017), and to distract from unwanted symptoms, for example addiction withdrawal (Goldenhersch et al., 2020), makes it particularly promising for clients who experience challenging psychedelic episodes, which may discourage them from revisiting these during the integration and self-practice sessions. Lastly, the process of self-practice does not need to be restricted to the laboratory, clinical or retreat environments (Bell et al., 2020), allowing a continued integration to take place at a local VR studio, VR clinic, or even at home, thanks to increasingly affordable technological advancements in the field.

### Rescue

Challenging experiences seem to play an important role in the psychedelic experience (Johnstad, 2021). Immersing in them instead of avoiding them seems to be the most effective way to diminish the anxiety or panic that they may otherwise cause and to extract the maximum benefit from the integration process (Carbonaro et al., 2016). Conversely, resisting the difficult experience could obstruct the therapy’s beneficial effects, impact the resolution of the issue and catalyze the progression from a challenging experience to an adverse effect (Vollenweider and Kometer, 2010). Therapists face difficulty in distinguishing when they should encourage leaning into that challenge, and when it may become detrimental to the participant and/or the therapist (Carbonaro et al., 2016). Currently, no tool exists that would be capable of terminating a potentially adverse progression both rapidly and transiently. One technique utilized, taking blindfolds off and performing relaxation exercises or similar, is a transient but not a reliable method. On the other hand, pharmacological interventions, like benzodiazepines, put an end or create an irreversible disruption to the experience and as a result, any therapeutic effects (Rey et al., 1999; Lerner et al., 2003; Bounds and Nelson, 2020).

In situations that impose acute emotional strain, VR is significantly more reliable at overriding stress responses than current alternative methods, including two-dimensional visual displays of similar scenarios (e.g., Riches et al., 2021). When applied during an acutely stressful situation, VR has already been shown to lead to immediate and significant lowering of stress levels, marked by changes in heart rate variability and cortisol levels, as well as significant improvements in subjective measures of anxiety and affect (Liszio et al., 2018). The strongly absorbing nature of immersive VR environments that are designed to act as an immediate anxiety relief (Valtchanov, 2010; Yu et al., 2020), offer a competing stimulus (Wiederhold et al., 2014; Nordgård and Låg, 2021) and may act as a sobering tool during an over-challenging or otherwise psychologically detrimental psychedelic experience. Even when not utilized, the knowledge that VR is available as an immediate rescue tool may act as a safety net for the participant, aiding the sense of relaxation, trust and surrender into the experience.

To act as a rescue tool, the VR scenario needs to compete with the strongly engaging inner experience. Therefore, a rescue VR tool should be characterized by a very rich contextual design; immersive sensory stimuli; dynamic, attention-demanding content and an interactive component, closely resembling a typical gaming environment. To act in opposition to the emotionally engaging therapeutic process, the audio content of a rescue VR scenario should also contrast any musical input applied up to that point. VR will thus act as a purposeful distractor from the internal experience, possibly changing the quality of the experience dramatically and irrevocably. Therefore, it is critical that the decision to use it is not made lightly.

## Limitations of Virtual Reality and Guidelines for Safety

### Over-Stimulation

Virtual reality allows a wide range of design alternatives, with an abundance of lights, colors, shapes, motion features and interactivity options to select from Ewalt (2018). Some designs can be strongly stimulating, even to the point of visual discomfort (Saredakis et al., 2020), and the potential risk of over-stimulation (and in extreme cases, seizures) has been raised by VR providers (Oculus, 2020). Care needs to be taken around combining any powerful, external stimulus with the already intense sensory experiences brought on by a psychedelic substance. VR content provided to participants should avoid sudden changes in visual patterns, extremes of saturation or brilliance and spatial disorientation. Additionally, although adverse events of VR that involve seizures have not been documented, even in people with known photosensitive epilepsy (Tychsen and Thio, 2020), protocols that combine the use of VR and psychedelics should exclude individuals that may be at risk of epilepsy, until there is greater understanding of this potential adverse effect.

### Accidental Exposure

A traumatic response can be triggered unexpectedly by any relevant cue (Van der Kolk, 1998). Highly immersive VR environments can easily act as a trigger, if their content is directly related to the traumatic experience (Difede, 2016; Rizzo et al., 2017), particularly during the vulnerable state induced by psychedelic substances (Carhart-Harris et al., 2018). Unless exposure is the intention of treatment, the VR content needs to be examined for presence of any cues that have personal resonance or could be associated with traumatic experiences, both at a generic and individual level. Generically, this should be achieved by considering and maintaining care around common triggers, for example heights, dark spaces, narrow or enclosed spaces, scenarios in or under water, etc. At an individual level, key triggers need to be discussed with the participant during preparation and, if possible, the VR scenario should first be examined when sober, if it is later to be used when under the influence of the psychedelic substance.

### Leading

The expectancy effects associated with psychedelics mean that selection of VR environments will have a strong impact on the psychedelic journey (as do environments in the real world; Carhart-Harris et al., 2018). Therefore, introducing any VR input, especially immediately prior to dosing or during the pre-peak or peak phases, must be treated with utmost care. A delicate balance needs to be struck between providing mild stimuli to block distraction from mundane external realities and maintaining focus on the intention (guiding), whilst avoiding hijacking of the experience and dictating its content (leading). The use of symbols and cues, for example to strengthen the intention, if necessary, needs to be carefully weighed against the risk of overtaking/dictating the experience completely. As such, VR may be best avoided as the first line of guidance in the particularly vulnerable parts of the journey (pre-peak and peak); instead, being better suited for patients who received psychedelic treatment previously, but gained little benefits from it, or relapsed. Limited response to PP may be caused by avoidance, anxiety or lack of clarity around intention (Carbonaro et al., 2016; Davis et al., 2020), which are the areas of care that VR may be able to assist with.

### Distraction

In many PP protocols, external stimulation during the dosing session is kept to minimum, and the participant is encouraged to focus their attention inward, as any sensory input may cause disruption to the unfolding internal narrative. Therefore, potential benefits of utilizing VR during dosing, including peak and post-peak phases, have to be carefully weighed against potential disadvantages. There is currently no evidence to suggest that VR may be of added benefit during the peak phase. VR may be best reserved for the post-peak phase of the experience, when most of that internal narrative has unfolded. Nonetheless, it is critical to avoid overly leading this process as an active engagement with VR may lead to some participants feeling urged to verbalize their experience before they are ready to do so. This may lead to vague or even misguided conclusions therefore the process of recall and sense making should not be rushed. Additionally, explicit imagery or journey-based scenarios may replace the internal insights. The use of personalized, patient-driven design is therefore critical. Lastly, as outlined in the rescue scenario, interactive VR content is likely to compete with the effects of the drug and should only be used as a rescue function. Otherwise, cognitive interaction, for example *via* task-oriented activities (da Costa et al., 2021) or physical interaction, for example *via* the use of controllers or real-life movement (Lee et al., 2015), may be best avoided during dosing, as these may be particularly impactful in distracting from attention to the internal state. Even in the case of intentional use of VR as a distracting stimulus, it should not be hastily applied to “rescue” participants during challenging psychedelic experiences but should be reserved for when the therapist assessment is of impending progression to an adverse experience.

### Cyber-Sickness and Physical Discomfort

The physical comfort of the patient needs to be considered. VR stimulus can lead to simulator sickness symptoms, most commonly fatigue, headache and nausea, (Akiduki et al., 2003; Norman, 2018), which may be caused by poor coordination between the visual VR stimulus and the real life movement, creating the so called “sensory conflict” (Dużmańska et al., 2018; Weech et al., 2019). Although most simulator sickness symptoms are mild and resolve quickly (Nichols and Patel, 2002), they may be exacerbated by the simultaneous occurrence of gastrointestinal effects of psychedelics (Johnson et al., 2012; Reiff et al., 2020). Cyber-sickness symptoms can be mitigated with careful consideration of VR design specifications, for example: avoiding controller-based movements, minimizing gaming content in favor of scenic or minimalistic content (Saredakis et al., 2020), or maintaining an appropriate exposure time (Dużmańska et al., 2018). Finally, the head mounted visual display, technical glitches or poor visual quality of the VR model may cause some discomfort (Norman, 2018), which may be of particular relevance when under the influence of a psychedelic substance, when senses are heightened and uncomfortable bodily sensations may already be present (Reiff et al., 2020). Therefore, attention needs to be devoted to using the highest quality content and equipment as well as adjusting the VR set-up for maximum comfort of the participant, including for participants who wear glasses. Individuals with severe visual impairment or who wear large glasses that cannot be fitted into the VR headset may need to be excluded from treatment.

### Resource Limitation

Introduction of VR technology into therapy requires the development of novel protocols and procedures as well as training of practitioners (McMahon and Boeldt, 2021). Therapists would be required to upskill to take advantage of VR’s unique features and, more importantly, to ensure that any VR-related technological malfunctions or psychological adverse reactions are mitigated. Additionally, VR becomes a new variable that the therapist needs to control, potentially distracting them from being fully present for the patient. A large dose of comfort, familiarity and control over the VR software and hardware is required before it can be employed by the practitioner, which puts additional demand on training time and resources.

### Commercial Interests

Virtual reality is currently predominantly used as entertainment technology (Cipresso et al., 2018). Its use in therapy is largely unregulated and numerous VR products that target therapeutic, or well-being applications have not been scientifically tested or validated. Such validation is of particular importance when technology is used in combination with psychoactive substances that can alter processing of external and internal stimuli as profoundly as psychedelic compounds. An increasing interest in introducing VR into PP calls for an urgent need for careful evaluation, ideally *via* a scientific, peer-reviewed process. Additionally, VR models should always be supplied with robust protocols that consider application, timing, technical requirements, mitigation of potential side effects and response to adverse events, among others.

## Summary

Psychedelic-assisted psychotherapy presents itself as a promising and attractive alternative to established psychiatric treatments (Nichols, 2016; Belouin and Henningfield, 2018). The recent revival in research on therapies that use psychedelics highlights a unique set of benefits, along with a unique set of challenges and limitations of this approach (Wheeler and Dyer, 2020; Williams et al., 2021). In parallel, VR is reportedly one of the most enjoyable and comfortable forms of therapy (Garrett et al., 2017), having one of the highest compliance rates and best therapeutic alliance across all forms of treatments (Meyerbröker and Emmelkamp, 2008; Wilson et al., 2008), even among individuals resistant to other treatments (Riva et al., 2019). It can be used to provide the most favorable environmental stimulus with a high level of control and thus reinforce PP with properties in which current protocols may be inadequate.

In supporting treatments that use psychedelics, VR can be utilized for its ability to:

1. Mitigate psychological side effects through enhancing the state of relaxation,
2. Help participants sustain their focus on intention by removing familiar cues that keep them tethered to their external world,
3. Encourage entering the inner world of experience by inducing a mindful presence,
4. Deepen the intensity of acute psychological and emotional states *via* simultaneous targeting of ME-evoking pathways,
5. Prime the capacity to achieve an ASC through familiarization and comfort with the ASC experience,
6. Enhance and maintain a hierarchy-free therapeutic alliance that is consistent throughout treatment,
7. Strengthen resilience and a sense of agency around facing challenging experiences.

To maximize potential benefits from profound ASC experiences, including ME, VR can be used to prime occurrences of those states by training the capacity to enter them during preparation; augment their depth and facilitate their profound emotional impact during dosing; and enhance therapeutic utility by aiding revisiting of these states during integration. When working in synergy with changing features of the psychedelic experience, VR scenarios can offer gentle guidance during the delicate process of transition from normal to altered consciousness and from altered to normal consciousness (Fox et al., 2018). When used repeatedly across multiple, different PP sessions, VR can provide continuity to the experience of treatment, from preparation to dosing, from dosing to integration and from integration to self-practice. Further, VR may extend the benefits of PP by enabling and encouraging deeper exploration of insights that emerged during integration, and inspire continued, self-paced practice of beneficial activities, for example meditation or relaxation (Mithoefer et al., 2008). Last but not least, the VR world is a safe, comfortable and intuitive intervention that, when needed, can temporarily distract from an overly challenging or adverse experience, or disrupt the process of being stuck in an experiential loop without terminating treatment.

Finally, it is noted that special care needs to be taken when using VR as a part of psychedelic psychotherapy, particularly around not introducing potentially disturbing or traumatic triggers, distracting from the inner experience, leading the narrative of the psychedelic experience or providing overstimulating or cyber-sickness inducing content. We recommend that any VR scenario that may be introduced into PP is developed in accordance with a robust protocol, both of which are scientifically validated and accompanied by thorough training of any practitioners involved in therapy.

## Conclusion

We described features of VR that make it a promising candidate as a complementary moderator of therapies that utilize psychedelic substances. Whilst these features are yet to be empirically explored in relation to psychedelics, we propose a range of potential synergistic applications of VR and PP and evaluate their individual advantages and disadvantages aiming to inspire an informed research practice in this emerging field. We suggest that a comfortable, adaptable, and reliable VR setting may support treatment with psychedelics *via* mitigating adverse psychological states, catalyzing the effects of each phase of the psychedelic experience and building a cohesive trajectory for the entire PP. Further research could explore the potential application of VR to: expand and deepen mystical and peak experiences; guide transition into and out of an altered state of consciousness; promote a continuous, multi-sensory experience through the entire psychedelic journey; and offer a non-invasive mitigation of adverse events. These potential synergistic applications need to be empirically validated in the view of potential limitations prior to commercial application.

## Author Contributions

AS was in charge of overall direction and planning of the manuscript, collected, analyzed, and synthesized the evidence, created the first and main conceptual themes of the manuscript, and drafted the first version of the manuscript, that was then further re-examined conceptually together with PP, as new evidence was explored. AS and PP explored those conceptual themes and prepared the manuscript outline. PP refined the manuscript and the language. LD was responsible for supervising the process and critical revision of the manuscript. All authors contributed and approved the final version of the manuscript.

## Conflict of Interest

AS and PP are co-founders of Enosis Therapeutics Pty. Ltd., a self-funded commercial venture that specializes in the design of VR environments for the psychedelic experience. The remaining author declares that the research was conducted in the absence of any commercial or financial relationships that could be construed as a potential conflict of interest.

## Publisher’s Note

All claims expressed in this article are solely those of the authors and do not necessarily represent those of their affiliated organizations, or those of the publisher, the editors and the reviewers. Any product that may be evaluated in this article, or claim that may be made by its manufacturer, is not guaranteed or endorsed by the publisher.
